# Learnable frozen feature augmentation for few-shot biomarker prediction from pathology whole-slide images

**DOI:** 10.1093/bioinformatics/btag597

**Published:** 2026-08-07

**Authors:** Di Zhang, Jiashuai Liu, Youyuan Ma, Jiusong Ge, Zhi Zeng, Wenfang Sun, Qidong Liu, Kai He, Yefeng Zheng, Weimiao Yu, Chen Li, Zeyu Gao

**Affiliations:** School of Computer Science and Technology, Xi’an Jiaotong University, Xi’an 710049, China; School of Computer Science and Technology, Xi’an Jiaotong University, Xi’an 710049, China; School of Computer Science and Technology, Xi’an Jiaotong University, Xi’an 710049, China; School of Computer Science and Technology, Xi’an Jiaotong University, Xi’an 710049, China; School of Computer Science and Technology, Xi’an Jiaotong University, Xi’an 710049, China; Informatics Institute, University of Amsterdam, Amsterdam 1098 XH, The Netherlands; School of Computer Science and Technology, Xi’an Jiaotong University, Xi’an 710049, China; Saw Swee Hock School of Public Health, National University of Singapore, Singapore 119077, Singapore; Medical Artificial Intelligence Laboratory, Westlake University, Hangzhou 310030, China; Bioinformatics Institute, Agency for Science, Technology and Research (A*STAR), Singapore 138671, Singapore; School of Computer Science and Technology, Xi’an Jiaotong University, Xi’an 710049, China; Department of Oncology, University of Cambridge, Cambridge, CB2 0XZ, United Kingdom

## Abstract

**Motivation:**

Whole-slide image (WSI)-based biomarker prediction in computational pathology has the potential to support scalable and resource-efficient analysis of large pathology cohorts, helping prioritize cases for downstream molecular testing and patient stratification. However, reliable biomarker labels are often limited and costly to obtain, making label-efficient WSI prediction essential. Recent slide-level foundation models have opened new opportunities for few-shot biomarker prediction by providing strong pretrained slide representations. Nevertheless, few-shot learning still suffers from sparse labeled support data, and data augmentation remains important for improving robustness and generalization. In frozen multi-stage WSI pipelines, however, conventional augmentation is difficult to apply: pixel-level augmentation requires costly feature re-extraction, while naive perturbation of frozen representations may compromise semantic consistency.

**Results:**

To address this challenge, we propose Learnable Frozen Feature Augmentation (LFFA), a training-time feature-space augmentation framework for few-shot WSI biomarker prediction. Instead of directly perturbing frozen slide features, LFFA learns controllable augmented slide views from contextualized interaction tokens under geometry-aware and downstream-supervised constraints, improving representation diversity while preserving semantic consistency. The augmented features are further optimized with an augmented-class α-mix loss to balance diversity and class semantics. We evaluate LFFA on three few-shot WSI biomarker prediction tasks, covering molecular marker prediction and gene mutation prediction, using three representative and widely used slide-level foundation models. Results show that LFFA consistently improves the corresponding baselines and achieves stronger overall robustness than existing augmentation methods across tasks, backbones, and shot settings.

**Availability and implementation:**

Source code and processed experimental splits will be made available at https://github.com/zdipath/LFFA.

## 1 Introduction

Computational pathology (CPath) aims to extract clinically meaningful information from whole-slide images (WSIs) to support clinical decision-making (Bilal *et al.* 2025, Li *et al.* 2025, Zhang *et al.* 2025). Biomarker prediction from pathology images is clinically important because it can support molecular profiling, treatment stratification, and prognostic assessment, particularly in cancer where molecular status often guides therapeutic decisions (Liu *et al.* 2025). However, the labels for biomarkers associated with pathology images are difficult to obtain due to the high cost and complexity of the diagnostic tests required to identify these biomarkers (He *et al.* 2026). This data scarcity makes few-shot learning an essential approach, as it allows models to adapt from a small number of labeled WSIs to effectively predict biomarkers across diverse patient populations.

Few-shot WSI classification has been actively studied through vision-language modeling, multiple instance learning (MIL)-based adaptation, and prototype calibration (Quan *et al.* 2024, Shi *et al.* 2024, Guo *et al.* 2025). However, these methods still rely on learning task-specific adaptation or aggregation modules from very limited weak slide-level labels, which can hinder generalization (Li *et al.* 2023) and increase overfitting risk in the low-data regime (Farina *et al.* 2025). Meanwhile, recent slide-level FMs (Shaikovski *et al.* 2024, Xu *et al.* 2024, Ding *et al.* 2025, Zhang *et al.* 2026) are pretrained on large-scale WSI corpora and provide strong slide-level representations. Even when used as frozen feature extractors, they enable a more practical feature-extraction-and-caching workflow for few-shot classification. This frozen feature-extraction and caching workflow is practically attractive because slide-level features can be extracted once and reused across downstream few-shot tasks. Considering their brilliant performance, the key challenge is often not to further increase model flexibility, but to better exploit the few available labeled slides, which usually cover only a limited fraction of each class’s morphological variability. This naturally motivates a complementary data-centric question: *can we better exploit the few available labeled slides by enriching their representations, without modifying the pretrained slide-level encoder?*

**Figure 1:**
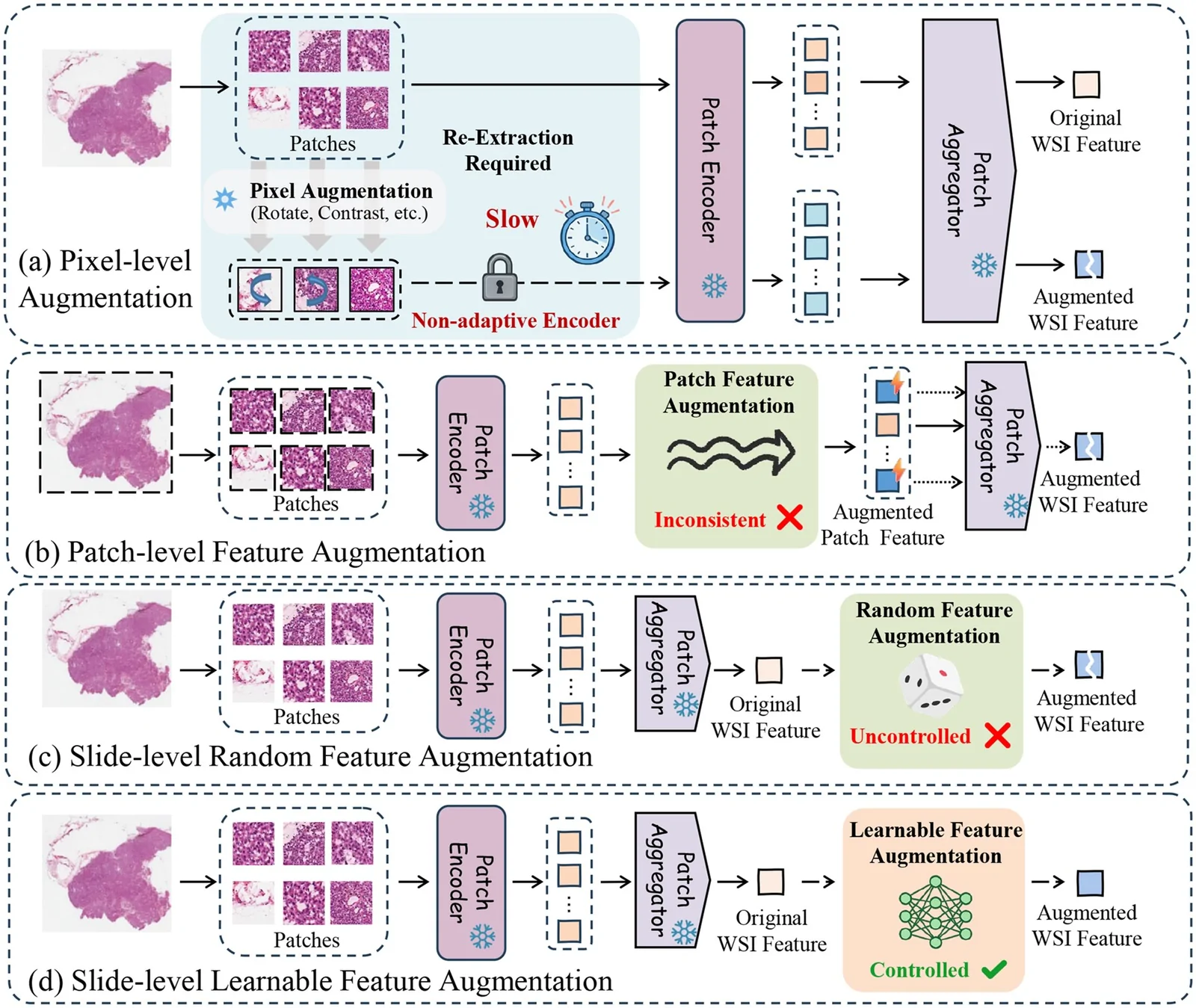
Motivation and comparison of augmentation strategies for few-shot WSI learning. (a) Pixel-level augmentation requires re-extracting patch features and re-aggregating WSIs. (b) Patch-level perturbation is difficult to translate into stable slide representations under frozen aggregation. (c) Random perturbation of frozen representations introduces uncontrolled feature variation in few-shot training. (d) LFFA performs controlled feature-space augmentation with a lightweight module to generate label-consistent feature variants.

A natural route is augmentation, which is widely used in few-shot learning to alleviate overfitting and improve generalization (Mumuni and Mumuni 2022, Zheng *et al.* 2025). However, as illustrated in Fig. 1a, pixel-space augmentation is poorly suited to frozen slide-level FM pipelines: it requires re-extracting patch features and re-aggregating WSIs, thereby breaking the efficient cache-and-reuse workflow, while the frozen encoder cannot adapt to absorb invariances induced by pixel perturbations. This motivates moving augmentation into representation space. Moreover, directly perturbing large numbers of patch- or instance-level features is problematic under the frozen aggregation or encoding setting in Fig. 1b, because local perturbations are difficult to propagate into a stable and label-preserving slide representation. This limitation affects most existing WSI-specific augmentation methods, which operate at the patch or instance level and typically assume a trainable MIL aggregator (Yang *et al.* 2022, Gadermayr *et al.* 2023). It is even more restrictive for methods that require an additional trainable generator or teacher model (Chen and Lu 2023, Zaffar *et al.* 2023, Tang *et al.* 2025), as such requirements conflict with the frozen, cache-and-reuse few-shot setting targeted in this work. As shown in Fig. 1c, randomly perturbing frozen slide-level representations is difficult to control and can produce noisy or label-inconsistent variants, which is particularly problematic in the few-shot regime (Bär *et al.* 2024, Ling *et al.* 2025). These observations suggest that augmentation in frozen WSI pipelines should be learned from downstream supervision rather than defined by random perturbations, while remaining controlled to preserve semantic consistency under scarce biomarker labels.

To address these challenges, we propose *Learnable Frozen Feature Augmentation (LFFA)*, a training-time augmentation framework for few-shot WSI prediction with frozen slide-level foundation models. LFFA improves model performance by leveraging *Contextualized Interaction Tokens (CITs)*—token-level representations that remain in the same feature space as the slide embedding while preserving finer-grained semantics. Unlike raw patch features, which are difficult to control in frozen aggregation, or the final slide vector, which is too coarse for structured variation, CITs offer a more suitable entry point for augmentation. LFFA applies controlled perturbations to the CITs, ensuring that the augmentations introduce local variation while preserving semantic consistency. It then lifts these token updates into slide space through a geometry-aware prior proposal followed by multi-head attention refinement, producing more discriminative and label-consistent augmented representations. An augmented-class α-mix loss is introduced to balance diversity and consistency, ensuring the generated views remain class-preserving without collapsing into trivial copies of the original features.

To evaluate LFFA, we construct a few-shot WSI classification benchmark spanning three tasks across three slide-level foundation models, namely TITAN (Ding *et al.* 2025), PRISM (Shaikovski *et al.* 2024), and GigaPath (Xu *et al.* 2024). Experiments show that LFFA consistently improves few-shot classification over the corresponding baselines and achieves stronger overall robustness than existing feature-level augmentation methods across tasks, backbones, and shot settings. Our contributions are summarized as follows:

- We formulate few-shot WSI prediction under frozen slide-level foundation models as a cache-efficient adaptation setting, and propose *LFFA*, a training-time feature-space augmentation framework that enriches limited labeled examples without modifying the pretrained slide-level encoder.
- We develop a controlled feature augmentation mechanism based on *CITs*, which learns non-trivial yet label-consistent augmented views through token-level perturbation, downstream-supervised slide-level refinement, and an augmented-class α-mix loss.
- We validate LFFA on few-shot WSI biomarker prediction, including molecular marker and gene mutation prediction, across three slide-level foundation models and three few-shot settings. LFFA consistently improves baseline performance, demonstrating its value for robust and label-efficient biomarker prediction.

## 2 Methodology

### 2.1 Problem setting

**Figure 2:**
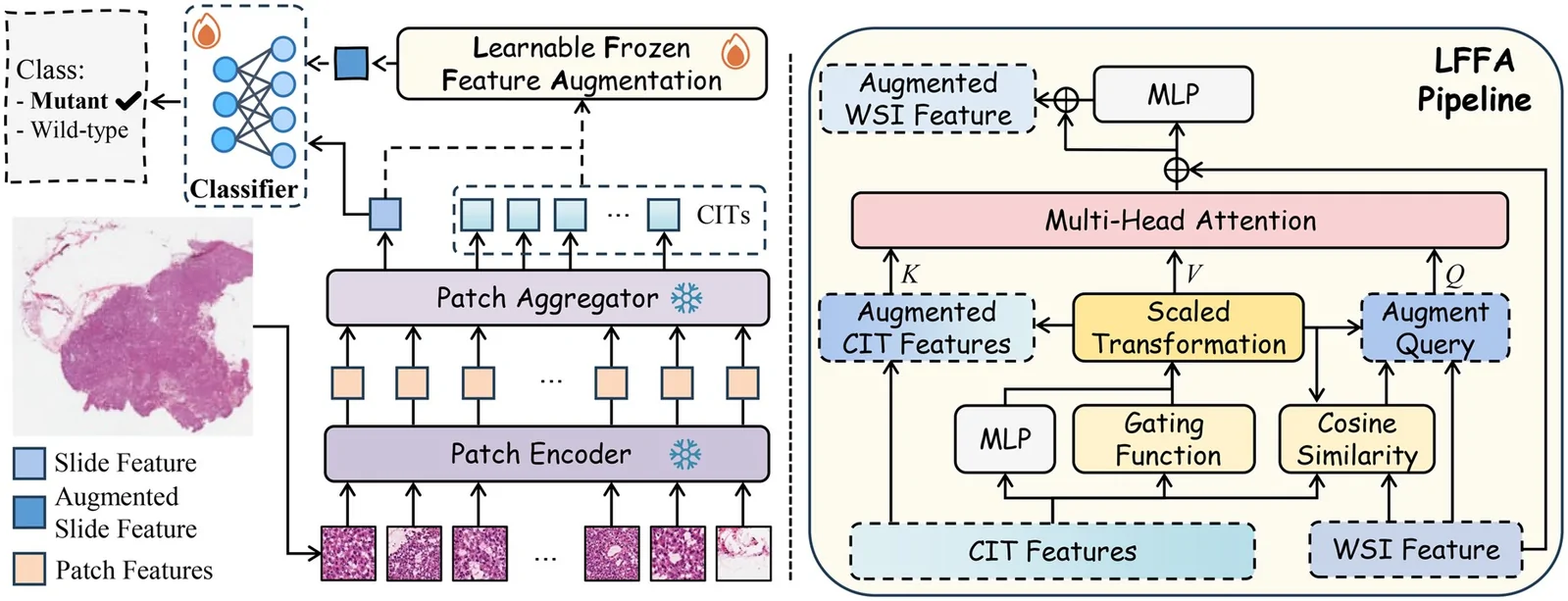
Overview of the proposed LFFA framework. **Left**: A typical multi-stage WSI pipeline, where the patch encoder and aggregator are frozen to produce a cached slide feature and CIT features. **Right**: LFFA applies learnable gated perturbations to CITs, then lifts the token updates to slide level to form an augmented slide feature for downstream classification.

As shown in the left part of Fig. 2, computational pathology typically relies on a multi-stage WSI pipeline rather than end-to-end modeling, due to the extremely high resolution of WSIs. A slide is first tessellated into image patches, which are encoded by a patch-level foundation encoder and then aggregated by a slide-level foundation model to produce slide-level representations for downstream prediction.

Firstly, given a WSI *X*, we tessellate the tissue region into a set of image patches ${\left\{x_{i}\right\}}_{i=1}^{M}$, where *M* denotes the number of extracted patches and each patch $x_{i}\in R^{p\times p\times 3}$ corresponds to a local field of view of spatial size p×p. Secondly, each patch is encoded by a patch-level foundation encoder $f_{\theta }$, a pretrained extractor to obtain a compact feature representation $h_{i}=f_{\theta }(x_{i})\in R^{d}$, where *d* is the feature dimension and i=1,…,M. Finally, a slide-level FM aggregates the patch feature set $H={\left[h_{1},h_{2},\ldots ,h_{M}\right]}^{⊤}\in R^{M\times d}$ into a global slide representation for downstream prediction. We denote the aggregation operator by A and write its output as (s,T)=A(H), where $s\in R^{d}$ is the slide embedding used for classification or regression, and $T\in R^{K\times d}$ denotes *K* additional token-level outputs produced by the aggregator.

We refer to *T* as CITs, i.e., token-level outputs that lie in the same feature space as the slide embedding *s* while retaining finer-grained semantic information. For TITAN and GigaPath, CITs are contextualized tokens computed from patch embeddings, whereas for PRISM they are latent tokens generated by learnable queries. These frozen outputs provide the basis for our feature augmentation framework. As illustrated in the right part of Fig. 2, LFFA operates on CITs and lifts the resulting token updates to slide space to form an augmented slide embedding for downstream prediction.

### 2.2 Learnable CIT augmentation

Directly augmenting the slide embedding *s* can be unreliable. As a highly compressed global summary, *s* offers limited controllability: naive perturbations may inject noise and violate semantic consistency. We therefore perform augmentation at the token level using CITs, which carry comparable semantic information at finer granularity. Augmenting CITs provides a more controllable way to enrich the slide representation, enabling more faithful slide-level enhancement in later stages.

To make token augmentation more controllable and semantically consistent, we decompose the perturbation of each CIT into two complementary factors: direction and magnitude. Specifically, for the *i*-th token $t_{i}$, we predict an update direction by $d_{i}=\text{MLP}(t_{i})$, where MLP(·) is a two-layer feed-forward network with the GELU activation function and dropout. We further estimate a scalar gate $g_{i}=\sigma (w^{⊤}t_{i}+b)$ to control the perturbation magnitude and suppress unreliable token updates. The augmented token is then given by $t_{i}^{\text{aug}}=t_{i}+\Delta t_{i}$, where the perturbation is defined as $\Delta t_{i}=g_{i}d_{i}$.

Because even small misaligned updates can accumulate into semantic drift in the few-shot setting, token perturbations should remain explicitly bounded. We therefore use the sigmoid gate $g_{i}\in (0,1)$ as a lightweight regulator: it provides a natural “skip” behaviour for unreliable tokens, while allowing larger updates only when the token context suggests a beneficial modification.

### 2.3 Task-specific WSI augmentation

After obtaining augmented CITs, we lift token-level changes to a slide-level augmentation in two stages: a *geometry-aware prior proposal* induced by the backbone’s representation geometry, followed by a *task-specific refinement* driven by downstream supervision.

*Geometry-aware prior proposal*. We first compute token weights by measuring each augmented token’s semantic alignment with the slide embedding via cosine similarity,


(1) $$a_{i}=\langle \frac{t_{i}^{\text{aug}}}{\left\|t_{i}^{\text{aug}}\right\|},\frac{s}{\left\|s\right\|}\rangle ,\;i=1,\ldots ,K,$$

and transform these scores into a normalized distribution through a temperature-scaled softmax,


(2) $$w_{i}=\frac{\;\text{exp}(\gamma a_{i})}{\sum_{j=1}^{K}\;\text{exp}\;(\gamma a_{j})},$$

where γ controls the sharpness. Using these weights, we inject a similarity-weighted summary of token updates into the slide embedding to form an augmentation query


(3) $$q=s+\sum_{i=1}^{K}w_{i}\Delta t_{i}\in R^{d}.$$

Since *q* is determined solely by the frozen backbone’s feature relations between *s* and $t_{i}^{\text{aug}}$, it serves as a geometry-aware augmentation proposal, which we refer to as the WSI’s *prior augmentation*.

*Task-specific refinement.* The frozen prior alone can be suboptimal for a specific downstream task, as the same WSI representations are shared across diverse objectives. We therefore refine it by retrieving a slide-conditioned augmentation signal from the augmented tokens via multi-head attention. Let $T^{\text{aug}}={\left\{t_{i}^{\text{aug}}\right\}}_{i=1}^{K}\in R^{K\times d}$ be the augmented CITs used as keys, and $\Delta T={\left\{\Delta t_{i}\right\}}_{i=1}^{K}\in R^{K\times d}$ be the corresponding updates used as values. We compute


(4) $$s^{\text{att}}=s+\text{MHA}(q,T^{\text{aug}},\Delta T),$$

where MHA is multi-head attention, and pass $s^{\text{att}}$ through a residual connection to obtain the final slide-level augmentation:


(5) $$s^{\text{aug}}=\text{MLP}(s^{\text{att}})+s^{\text{att}}.$$

Here, $s^{\text{aug}}$ can be viewed as a *task-specific augmentation*, as it re-weights and composes token-level updates conditioned on the downstream objective.

### 2.4 Augmented-class α-mix loss

A robust augmentation objective should balance two competing goals: (i) *diversity*, encouraging augmented representations to provide non-trivial variation beyond class prototypes, and (ii) *consistency*, preventing augmented samples from drifting outside the correct decision region.

To achieve this balance, we introduce an *augmentation-aware* objective that treats an augmented feature as a soft mixture of the original semantic label and an auxiliary augmentation indicator.

We implement the 2 *N*-way head as a cosine-prototype classifier. $W=[w_{1},\ldots ,w_{2N}]\in R^{d\times 2N}$ denotes the prototype matrix, where each column $w_{c}$ is an $\ell_{2}$-normalized class prototype in the embedding space. We also $\ell_{2}$-normalize features before classification, so $W^{⊤}s$ in Eq. (6) corresponds to scaled cosine similarities,


(6) $$p=\text{softmax}(W^{⊤}s),\;p^{\text{aug}}=\text{softmax}(W^{⊤}s^{\text{aug}}).$$

For the original representation, we use the standard cross-entropy loss $L_{\text{ori}}=-\text{log}\;p_{y}$ with the ground-truth label y∈{1,…,N}. For the augmented representation, instead of enforcing a hard label, we define a soft target distribution $q\in R^{2N}$ that assigns probability mass 1−α to the original class *y* and mass α to the corresponding augmentation indicator class N+y:


(7) $$q_{c}=\{\begin{array}{cc} 1-\alpha , & c=y,\\ \alpha , & c=N+y,\\ 0, & \text{otherwise}. \end{array}$$

We then minimize the cross-entropy between *q* and $p^{\text{aug}}$:


(8) $$\begin{array}{c} L_{\text{aug}}=-\sum_{c=1}^{2N}q_{c}\;\text{log}\;p_{c}^{\text{aug}}\\ =-(1-\alpha )\;\text{log}\;p_{y}^{\text{aug}}-\alpha \;\text{log}\;p_{N+y}^{\text{aug}}. \end{array}$$

This objective preserves semantic consistency by keeping most probability mass on the original label, while reserving α mass to explicitly accommodate augmentation-induced variation. Finally, we combine the two losses:


(9) $$L=(1-\lambda )L_{\text{ori}}+\lambda L_{\text{aug}},$$

where λ is a balancing coefficient.

*Inference*. LFFA is used only during training and is completely removed at inference. Given a cached frozen slide embedding *s*, we classify it using only the *N* original-class prototypes. The auxiliary prototypes and the entire augmentation branch are discarded.

To theoretically validate our method, we analyze the geometric properties of the augmented features. We provide formal proofs establishing both properties below.

**Definition 1.** We say that a feature representation $s_{t}$ exhibits *asymptotic margin explosion* during training if, as the number of optimization steps t→∞, the logit margin between the target class *y* and any other class *j* diverges to infinity:


(10) $$\underset{t\to \infty }{\text{lim}}\left(w_{y}^{⊤}s_{t}-\underset{j\neq y}{\max}w_{j}^{⊤}s_{t}\right)=+\infty ,$$

where $w_{c}$ denotes the class prototype corresponding to class *c*, i.e., the *c*-th column of the weight matrix *W*.

**Theorem 1.** Consider the gradient flow dynamics driven by the augmentation loss, i.e., $\frac{ds^{\text{aug}}}{dt}=-\nabla_{s^{\text{aug}}}L_{\text{aug}}$. The optimization equilibrium enforces a strict upper bound on the logit margin between the target class y and the auxiliary class N+y. Specifically, the margin converges to a finite constant determined by α:


(11) $$\underset{t\to \infty }{\text{lim}}\left(w_{y}^{⊤}s_{t}^{\text{aug}}-w_{N+y}^{⊤}s_{t}^{\text{aug}}\right)=\text{ln}\left(\frac{1-\alpha }{\alpha }\right)<\infty .$$

This result shows that minimizing $L_{\text{aug}}$ strictly prevents the augmented feature $s^{\text{aug}}$ from asymptotically collapsing onto the class prototype $w_{y}$, thereby maintaining a non-degenerate margin and preserving feature diversity.

**Theorem 2.**  *Let* $g(t)=\|\text{MHA}(q,T^{\text{aug}},\Delta T){\|}_{2}$ denote the magnitude of the learnable perturbation in Equation (4) under the gradient flow dynamics. For any parameter α in Equation (7), the magnitude converges to a finite stationary point $g^{⋆}$, which is bounded by the logit constraint:


(12) $$\underset{t\to \infty }{\text{lim}}g(t)=g^{⋆}<\infty .$$

The proofs of Theorems 1 and 2 are provided in the Appendix. Together, Theorems 1 and 2 characterize a theoretical “safe zone” for learnable augmentation: the augmented features remain diverse enough to regularize, while the perturbations stay bounded to preserve decision-region consistency.

## 3 Experiments

### 3.1 Settings

#### 3.1.1 Datasets

We evaluate our method on a diverse collection of WSI benchmarks spanning multiple organs, molecular targets, and classification settings, including BCNB (Xu *et al.* 2021), MUT-HET-RCC (Acosta *et al.* 2022), and CPTAC-CCRCC. Concretely, we consider three few-shot tasks: *BCNB-ER*, which predicts ER status from breast cancer WSIs; *MUT-SETD2*, which predicts SETD2 mutation status from renal cancer WSIs; and *Cross-MUT-PBRM1*, a cross-cohort transfer task in which models are trained and validated on MUT-HET-RCC and tested on CPTAC-CCRCC to predict PBRM1 mutation status.

#### 3.1.2 Backbones and baselines

We evaluate few-shot WSI learning with three representative foundation backbones: TITAN (Ding *et al.* 2025), PRISM (Shaikovski *et al.* 2024), and GigaPath (Xu *et al.* 2024). Each backbone is coupled with its backbone-specific patch feature extractor, which is kept frozen to follow the standard multi-stage WSI pipeline and to ensure fair comparisons. We then conduct a comprehensive study of our proposed LFFA alongside other augmentation paradigms on all three backbones, covering pixel-level augmentation, WSI-specific MIL augmentation, patch-level feature augmentation, and slide-level feature augmentation. Our baselines include two pixel-level augmentations (Contrast, Brightness) (Goceri, 2023), two WSI-specific MIL augmentation methods, MixUp-MIL (Gadermayr *et al.* 2023) and ReMix (Yang *et al.* 2022), two feature-level slide augmentation methods (FroFA (Bär *et al.* 2024, Ling *et al.* 2025) and FATL (Bai *et al.* 2025)), and their patch-level variants, denoted as P-FroFA and P-FATL. The latter randomly augment a proportion *K* of patch features using FroFA/FATL before frozen aggregation, with *K* tuned by validation. For MixUp-MIL and ReMix, we adapt their original MIL-oriented mixing strategies to the frozen-feature setting after feature extraction. This setup isolates the effect of augmentation strategies under identical few-shot protocols across heterogeneous WSI models.

#### 3.1.3 Implementation details

We follow the standard CLAM-style WSI workflow for patch tiling and patch-level feature extraction (Lu *et al.* 2021). For each backbone, we freeze all foundation model parameters, precompute the slide-level representations together with the corresponding CIT features, and save them to disk. Subsequent few-shot training and evaluation are conducted by loading cached feature files only, without re-reading WSI images, which enables fast iteration and inference. For pixel-level augmentation, we apply contrast (Pixel-Ctr) or brightness (Pixel-Bri) perturbations to the WSI image and then rerun the frozen pipeline to extract the corresponding augmented features for training. The downstream model is trained on the combined set of original and augmented WSI features. Data augmentation is applied only during training. All feature extraction, aggregation, and downstream training are performed on a single NVIDIA RTX 4090 GPU.

#### 3.1.4 Evaluation metrics

We use five-fold cross-validation for all tasks. In each fold, data are split into train/val/test = 60/20/20. For each *k*-shot setting, *k* samples per class are randomly selected only from the training set, resulting in a class-balanced few-shot set. We report the mean and standard deviation across the five folds for each *k*-shot setting (where k∈{1,2,4}). Balanced Accuracy (ACC) serves as our primary metric, and AUROC (AUC) is also reported.

### 3.2 Experiment results and discussions

To better align the evaluation with label-efficient biomarker prediction, we focus on three biomarker-oriented few-shot WSI tasks: BCNB-ER, MUT-SETD2, and Cross-MUT-PBRM1. As summarized in Table 1, LFFA consistently improves the corresponding *w/o Aug* baselines across TITAN, PRISM, and GigaPath, with particularly clear gains in AUC. On TITAN and PRISM, LFFA achieves the best performance in almost all shot-metric combinations, showing its ability to exploit limited biomarker labels with strong frozen slide-level representations. Notably, on TITAN, the 2-shot LFFA result even surpasses the 4-shot *w/o Aug* baseline in both ACC and AUC, highlighting its label efficiency. On GigaPath, LFFA achieves the best AUC at 1-shot and 4-shot and remains competitive at 2-shot, suggesting stable ranking performance for biomarker prediction.

**Table 1:** Few-shot WSI classification performance across foundation models on BCNB-ER, MUT-SETD2, and Cross-MUT-PBRM1.

FM	Shot	Metric	w/o Aug	Pixel-Ctr	Pixel-Bri	MixUp-MIL	ReMix	P-FroFA	P-FATL	FroFA	FATL	Ours
**TITAN**	**1-shot**	ACC	54.20 (0.25)	54.36 (0.25)	54.70 (0.25)	53.78 (0.31)	53.82 (0.27)	54.60 (0.29)	54.56 (0.29)	55.47 (0.24)	56.30 (0.21)	**56.57 (0.20)**
		AUC	59.24 (0.67)	59.12 (0.69)	59.18 (0.70)	58.26 (0.75)	58.36 (0.78)	59.22 (0.67)	59.19 (0.67)	59.81 (0.50)	59.22 (0.77)	**60.92 (0.55)**
	**2-shot**	ACC	58.10 (0.32)	58.01 (0.32)	57.43 (0.28)	57.36 (0.41)	56.68 (0.38)	57.65 (0.36)	57.69 (0.36)	58.29 (0.24)	57.50 (0.32)	**59.49 (0.21)**
		AUC	61.33 (0.82)	61.33 (0.81)	61.13 (0.82)	61.26 (0.85)	60.59 (0.86)	61.48 (0.70)	61.45 (0.70)	63.58 (0.33)	62.14 (0.68)	**65.18 (0.22)**
	**4-shot**	ACC	58.64 (0.13)	59.17 (0.14)	59.17 (0.14)	59.46 (0.20)	58.74 (0.21)	59.26 (0.16)	59.17 (0.16)	60.15 (0.20)	59.65 (0.20)	**60.87 (0.14)**
		AUC	63.74 (0.30)	63.63 (0.31)	63.52 (0.33)	63.28 (0.30)	62.92 (0.32)	63.71 (0.32)	63.68 (0.32)	64.59 (0.22)	64.52 (0.31)	**64.90 (0.28)**
**PRISM**	**1-shot**	ACC	52.00 (0.40)	52.06 (0.39)	52.11 (0.38)	52.02 (0.23)	52.07 (0.29)	51.74 (0.25)	52.15 (0.25)	51.50 (0.22)	51.01 (0.25)	**53.23 (0.13)**
		AUC	51.56 (0.93)	51.65 (0.91)	51.37 (0.92)	52.12 (0.86)	52.55 (0.77)	52.38 (0.86)	52.66 (0.85)	53.08 (0.62)	49.93 (0.78)	**54.96 (0.69)**
	**2-shot**	ACC	55.21 (0.58)	54.90 (0.64)	56.08 (0.36)	55.55 (0.38)	53.96 (0.45)	55.82 (0.41)	55.39 (0.31)	55.43 (0.40)	54.29 (0.59)	**57.00 (0.18)**
		AUC	55.90 (1.27)	55.77 (1.29)	57.19 (0.95)	57.83 (0.90)	57.08 (1.20)	57.84 (0.91)	57.81 (0.92)	58.73 (0.65)	55.17 (1.39)	**60.54 (0.60)**
	**4-shot**	ACC	58.06 (0.20)	58.23 (0.23)	**58.55 (0.22)**	56.65 (0.24)	56.63 (0.24)	57.83 (0.29)	57.51 (0.32)	57.36 (0.27)	58.06 (0.21)	58.33 (0.17)
		AUC	59.88 (0.64)	60.00 (0.65)	60.25 (0.60)	60.33 (0.50)	61.35 (0.47)	60.55 (0.52)	60.50 (0.55)	61.29 (0.49)	59.79 (0.70)	**62.25 (0.36)**
**GigaPath**	**1-shot**	ACC	50.11 (0.02)	50.14 (0.02)	50.16 (0.02)	50.16 (0.03)	50.87 (0.07)	50.17 (0.03)	50.17 (0.03)	**53.62 (0.33)**	52.22 (0.21)	52.54 (0.11)
		AUC	54.70 (0.29)	54.71 (0.29)	54.70 (0.29)	55.26 (0.38)	55.50 (0.40)	55.25 (0.38)	55.23 (0.38)	55.60 (0.52)	55.35 (0.40)	**56.20 (0.47)**
	**2-shot**	ACC	52.99 (0.25)	52.90 (0.23)	52.95 (0.24)	53.07 (0.20)	54.30 (0.36)	53.16 (0.20)	53.14 (0.19)	54.35 (0.27)	**55.07 (0.41)**	53.89 (0.23)
		AUC	57.77 (0.37)	57.76 (0.37)	57.73 (0.36)	58.09 (0.40)	58.49 (0.50)	58.11 (0.40)	58.12 (0.40)	57.35 (0.51)	**59.05 (0.44)**	58.68 (0.42)
	**4-shot**	ACC	54.98 (0.22)	55.01 (0.21)	54.97 (0.24)	55.15 (0.22)	55.14 (0.20)	55.28 (0.21)	55.25 (0.20)	56.04 (0.19)	55.97 (0.14)	**57.10 (0.21)**
		AUC	59.19 (0.35)	59.19 (0.35)	59.19 (0.36)	59.50 (0.30)	59.58 (0.28)	59.46 (0.30)	59.46 (0.30)	59.55 (0.28)	59.40 (0.29)	**60.24 (0.28)**

ACC and AUC are reported under 1/2/4-shot settings for each augmentation method. Results are averaged over the three benchmark tasks and reported as mean (standard deviation). The best results are highlighted in bold, and the second-best results are underlined.

The comparison among augmentation strategies further highlights the advantage of controlled slide-level feature augmentation. Pixel-space augmentations, including contrast and brightness perturbations, bring only marginal or inconsistent improvements, which is expected in frozen WSI pipelines because pixel-level changes require feature re-extraction and cannot be absorbed by the frozen encoder. The WSI-specific MIL augmentation baselines, MixUp-MIL and ReMix, provide additional comparisons with established WSI augmentation strategies. Although these methods can improve performance in some cases, their gains are less stable across backbones and shot settings. For example, MixUp-MIL and ReMix lead to negative average improvements on TITAN, modest gains on PRISM, and more visible but still limited gains on GigaPath. Patch-level feature augmentation methods, such as P-FroFA and P-FATL, avoid this recomputation but still perturb local features before frozen aggregation, making it difficult to ensure stable slide-level semantic changes. In contrast, LFFA constructs task-aware augmented slide representations from contextualized interaction tokens, leading to more consistent improvements under scarce supervision. Detailed results for each individual task are provided in the Appendix, including BCNB-ER, MUT-SETD2, and Cross-MUT-PBRM1.

This trend is further supported by the robustness analysis in Table 2. Across the 18 task-shot-metric combinations for each backbone, LFFA achieves the highest success rate and the largest average improvement over the *w/o Aug* baseline on all three foundation models. Specifically, LFFA improves over the baseline in 88.9%, 88.9%, and 83.3% of the combinations on TITAN, PRISM, and GigaPath, respectively, with average gains of +2.12, +2.28, and +1.48. These results indicate that the proposed augmentation is not limited to isolated favorable cases, but provides robust and broadly effective improvements for few-shot biomarker prediction from pathology WSIs.

**Table 2:** Success rate and average improvement over the *w/o Aug* baseline in few-shot WSI learning.

Method	TITAN		PRISM		GigaPath	
	Win	**Avg** Δ	Win	**Avg** Δ	Win	**Avg** Δ
Pixel-Ctr	55.6%	+0.06	55.6%	−0.00	44.4%	−0.01
Pixel-Bri	55.6%	−0.02	72.2%	+0.49	33.3%	−0.01
MixUp-MIL	27.8%	−0.31	66.7%	+0.32	61.1%	+0.25
ReMix	11.1%	−0.69	66.7%	+0.17	**83.3%**	+0.69
P-FroFA	44.4%	+0.11	61.1%	+0.59	66.7%	+0.28
P-FATL	44.4%	+0.08	66.7%	+0.57	66.7%	+0.27
FroFA	72.2%	+1.11	66.7%	+0.80	77.8%	+1.13
FATL	77.8%	+0.68	33.3%	−0.73	61.1%	+1.22
Ours	**88.9%**	**+2.12**	**88.9%**	**+2.28**	**83.3%**	**+1.48**

For each backbone, statistics are computed jointly over 18 task-shot-metric combinations (3 tasks × 3 shot settings × 2 metrics: ACC and AUC), including BCNB-ER, MUT-SETD2, and Cross-MUT-PBRM1. Win denotes the fraction of combinations in which a method outperforms *w/o Aug*, and Avg Δ denotes the corresponding average improvement over the baseline.

### 3.3 Ablation studies

To assess the contribution of each design choice in LFFA, we conduct an ablation study on the three biomarker-oriented tasks, including BCNB-ER, MUT-SETD2, and Cross-MUT-PBRM1. Following the main evaluation protocol, we report the average AUC across the three tasks under the 2-shot setting in Table 3. In the variant without CIT augmentation, learnable augmentation is applied directly to the slide-level feature instead of the CITs. The variants w/o gate $g_{i}$ and w/o map $d_{i}$ remove the corresponding signal-shaping components in token perturbation. The variant w/o prior aug. replaces the prior-guided augmentation with average pooling to obtain *q* in Equations (2) and (3), while w/o refinement directly uses the prior-guided feature *q* without downstream task-specific refinement.

**Table 3:** Ablation study of LFFA modules on BCNB-ER, MUT-SETD2, and Cross-MUT-PBRM1.

Variant	TITAN	PRISM	GigaPath
w/o CIT aug.	63.64 (0.32)	59.56 (0.69)	58.16 (0.42)
w/o gate $g_{i}$	63.97 (0.35)	60.47 (0.62)	58.30 (0.45)
w/o map $d_{i}$	63.83 (0.33)	60.35 (0.64)	58.33 (0.41)
w/o prior aug.	63.95 (0.37)	60.24 (0.60)	58.50 (0.42)
w/o refinement	63.67 (0.29)	59.52 (0.72)	57.88 (0.39)
LFFA	**65.18 (0.22)**	**60.54 (0.60)**	**58.68 (0.42)**

We report the average AUC (%) across the three tasks in the 2-shot setting.

Overall, the full LFFA achieves the best average AUC on all three foundation backbones, indicating that its components are complementary for few-shot biomarker prediction. Removing CIT-based augmentation leads to clear drops, especially on TITAN and PRISM, suggesting that CITs offer a structured space better suited for controllable augmentation than final slide representations. The refinement module is also important, as its removal gives the lowest or near-lowest performance across backbones, particularly on GigaPath, showing that geometry-aware priors require task-specific refinement. Removing the gate $g_{i}$, nonlinear mapping $d_{i}$, or prior-guided proposal further reduces performance in most cases. These results highlight the joint benefit of token-level perturbation and task-aware refinement under scarce biomarker labels.

## 4 Conclusion

In the work, we studied few-shot biomarker prediction from pathology WSIs using frozen slide-level foundation models. To address the inefficiency of pixel-space augmentation and the instability of naive feature perturbation, we proposed LFFA, a learnable feature-space augmentation framework built on contextualized interaction tokens. LFFA generates semantically consistent augmented slide representations through token-level perturbation, geometry-aware slide-level augmentation, downstream task-specific refinement, and an augmentation-aware training objective.

Experiments on three biomarker-based WSI tasks, three slide-level foundation models, and multiple few-shot settings show that LFFA consistently improves the corresponding baselines and achieves strong overall robustness among compared augmentation methods. These results suggest that learnable feature-space augmentation offers a practical way to better exploit limited biomarker labels, supporting more label-efficient and scalable WSI-based biomarker prediction.

## Data Availability

The datasets used in this study are publicly available from their original repositories. Detailed instructions for accessing the datasets, together with the processed experimental splits used in this study, are available in the project repository at https://github.com/zdipath/LFFA.
